# Echocardiographic phenotypes of diabetic myocardial disorder: evolution over 15 months follow-up in the ARISE-HF trial

**DOI:** 10.1186/s12933-024-02554-y

**Published:** 2025-01-13

**Authors:** Thomas H. Marwick, Carolyn Lam, Yuxi Liu, Stefano Del Prato, Julio Rosenstock, Javed Butler, Justin Ezekowitz, Nasrien E. Ibrahim, W. H. Wilson Tang, Faiez Zannad, Riccardo Perfetti, James L. Januzzi

**Affiliations:** 1 Baker Heart and Diabetes Institute, Melbourne and Menzies Institute for Medical Research, Hobart, Australia; 2 National Heart Centre Singapore and Duke-National University of Singapore, Singapore, Singapore; 3 Cardiology Division, Massachusetts General Hospital, Harvard Medical School, Boston, MA USA; 4 Interdisciplinary Research Center “Health Science”, Sant’Anna School of Advanced Studies, Pisa, Italy; 5 Southwestern Medical Center, Velocity Clinical Research at Medical City and University of Texas, Dallas, TX USA; 6 Baylor Scott and White Research Institute, Dallas, TX USA; 7 University of Mississippi, Jackson, MS USA; 8 Canadian VIGOUR Centre, University of Alberta, Edmonton, AB Canada; 9 Cardiology Division, Brigham and Women’s Hospital, Harvard Medical School, Boston, MA USA; 10 Department of Cardiovascular Medicine, Heart Vascular and Thoracic Institute, Cleveland Clinic, Cleveland, OH USA; 11 CIC Inserm and CHRU Nancy, Université de Lorraine, Metz, France; 12 Applied Therapeutics Inc., New York, NY USA; 13 Heart Failure Trials, Baim Institute for Clinical Research, Boston, USA; 14 Baker Heart and Diabetes Institute, 75 Commercial Road, Melbourne, VIC 3004 Australia

**Keywords:** Diabetic myocardial disorder, Biomarkers, Systolic dysfunction, Diastolic dysfunction

## Abstract

**Background:**

Diabetic myocardial disorder (DbMD, evidenced by abnormal echocardiography or cardiac biomarkers) is a form of stage B heart failure (SBHF) at high risk for progression to overt HF. SBHF is defined by abnormal LV morphology and function and/or abnormal cardiac biomarker concentrations.

**Objective:**

To compare the evolution of four DbMD groups based on biomarkers alone, systolic and diastolic dysfunction alone, or their combination.

**Methods:**

The Aldose Reductase Inhibition for Stabilization of Exercise Capacity in Heart Failure (ARISE-HF) trial was a Phase 3 randomised trial of an aldose reductase inhibitor in patients with well-controlled type 2 diabetes mellitus (T2DM). The 1858 potential participants (age 67 ± 7 years; 50% women) were screened for SBHF based on abnormal echocardiography or biomarkers (N-terminal pro-B-type natriuretic peptide ≥ 40 ng/L or high sensitivity cardiac troponin T ≥ 10 ng/L [women] and ≥ 16 ng/L [men]). Exercise capacity (peak VO_2_) was reduced in 669 with DbMD (age 68 ± 7, 50% women), and peak VO_2_ was reassessed at 15 months.

**Results:**

The 1463 (79%) participants with DbMD were allocated to four clusters; 907 (49%) showed *isolated elevation of cardiac biomarkers*, 301 (16%) with *systolic dysfunction/hypertrophy*, 162 (9%) with *diastolic dysfunction* and 93 (5%) comprised an *overlap cluster* (combined diastolic, systolic or LV geometric abnormalities). Reduced VO_2_ (< 75% predicted) was present in 669 (46%); 72% of those with both systolic and diastolic dysfunction, 56% of those with systolic dysfunction and LVH, 53% of those with diastolic dysfunction and 38% with biomarkers alone (p < 0.0001). In 669 patients followed over 15 months, there was a similar small decrement in VO_2_ in all groups.

**Conclusions:**

Among individuals with T2DM and SBHF, reduced functional capacity is most prevalent in those with multiple physiological disturbances. However, there was no difference between phenogroups in the evolution of exercise intolerance.

*Trial Registration*: ARISE-HF, NCT04083339.

**Supplementary Information:**

The online version contains supplementary material available at 10.1186/s12933-024-02554-y.

## Background

Of all patients with HF, the group with type 2 diabetes (T2DM) are at the highest risk of adverse outcomes [1]. Heart failure (HF) may be the most common cardiac complication of T2DM, and is the most under-recognized among cardiovascular diseases in this population [2]. Before patients present with symptomatic HF, they commonly have Stage B HF (SBHF)– evidenced by abnormal structure, function or biomarkers in patients with risk factors, but no symptoms or signs of HF [3]. Despite having minimal or no symptoms, these patients commonly show reduced exercise capacity [4]. Recognition of patients during this stage may provide an opportunity to initiate cardioprotective therapy, and thereby prevent or delay the onset of clinical HF. T2DM is more likely to lead to HFpEF than HFrEF [5], especially in individuals with well controlled BP and absence of major CAD. Although sodium/glucose co-transporter 2 (SGLT2) inhibitors [6] and glucagon-like peptide-1 (GLP-1) receptor agonists [7] have been shown to be effective in patients with T2DM and overt HFpEF, their value in Stage B heart failure (SBHF) is unproven.

There are multiple causes of HF in T2DM– ranging from diabetic myocardial disorder (DbMD) reported by Rubler et al. [8], to common HF etiologies including hypertension and coronary artery disease [9]. The variety of different mechanisms [10] might be expected to engender a variety of phenotypes, potentially with different prognostic and management implications. Indeed, diagnostic approaches for subclinical LV dysfunction include elevated cardiac biomarkers [11], as well as various echocardiographic phenotypes that have been differentiated in a previous cluster analysis [12], involving remodeling, systolic and diastolic dysfunction (DD) [13]. However, acute disturbances of myocardial function in response to hyperglycemia or hypertension may explain some of these findings - previous observational studies of T2DM phenotypes had inconsistent levels of metabolic control. In addition, the implications of the subgroups in follow-up have not been investigated.

The ARISE-HF Trial (Aldose Reductase Inhibition for Stabilization of Exercise Capacity in Heart Failure) [14] was a phase 3 randomized study which showed that an aldose reductase inhibitor (AT-001) was safe but did not prevent decline in cardiac functional capacity in individuals T2DM and SBHF, compared with placebo. Participants had imaging and biomarkers at baseline and 15 months, with well-controlled metabolism and blood pressure, and no ischemic heart disease. The ARISE-HF database therefore provides a well-phenotyped cohort to better understand the presence, expression and evolution of SBHF among individuals with T2DM. We hypothesized that; (1) the phenogroups of individuals with DbMD would reflect the severity of functional compromise at baseline, (2) the phenogroups would show differences in evolution over 15 months of follow-up.

## Methods

### Study design

 The study group comprised patients recruited to the ARISE-HF trial (NCT04083339), a Phase 3, randomized, placebo-controlled trial that sought whether an aldose reductase inhibitor (AT-001) could stabilize exercise capacity in adults with T2DM and DbMD. Details of the study design and methodology have been published [14]. In this trial, patients with T2DM, with no cardiac history or cardiac symptoms, were screened for DbMD with cardiac biomarkers and echocardiography for the detection of LV remodeling, or systolic or diastolic dysfunction. Entry was restricted to those with adequate glycemic and blood pressure control [14], to exclude these as a cause for cardiac functional responses. SBHF was identified in patients with risk factors, no symptoms or signs, and one of structural, functional or biomarker evidence, consistent with the uniform definition of HF [3].

In the baseline analysis, we compared the clinical and exercise characteristics of predefined phenogroups, including those with reduced exercise capacity. For the follow-up analysis, we considered baseline and follow-up data in all participants with reduced exercise capacity at baseline (including both groups on AT-001 and placebo). We assessed the evolution of exercise capacity over 15 months in the biomarker and echocardiographic phenogroups defined at baseline.

### Clinical features

The baseline clinical data included demographics, past medical history, medication use and questionnaires regarding health status (KCCQ-23, a modification of the Kansas City Cardiomyopathy Questionnaire for a study population without overt HF) [15] and activity (the Physical Activity Score for the Elderly, PASE score) [16]. In addition to standard laboratory tests (including estimated glomerular filtration rate [eGFR], albumin/creatinine ratio, serum lipids and hemoglobin A1c [HbA1c]), we obtained N-terminal pro-B type natriuretic peptide (NT-proBNP; subclinical LVD threshold < 40 ng/L) and high sensitivity cardiac troponin T (hs-cTnT; <10 ng/L for women and < 16 ng/L for men).

### Echocardiography

A complete 2-dimensional and Doppler echocardiogram was performed using a standard acquisition [17]. Measurement and interpretation were blinded to clinical characteristics and treatment allocation, and provided by a core laboratory. Systolic dysfunction was measured as LV ejection fraction [LVEF] (LVEF < 40% was an exclusion for the trial), and global longitudinal shortening strain (GLS, abnormal < 16%) [18]. LV hypertrophy was defined by LV mass indexed for body surface area (LVMi *≥* 95 g/m^2^ and 115 g/m^2^ in women and men, respectively). DD was based on abnormal left atrial volume (LAVi > 34 mL/m^2^), increased ratio of early transmitral Doppler flow [E] over the early septal annular velocity [e’] (≥ 13), or estimated pulmonary artery pressures (right ventricular systolic pressure; RVSP > 35 mm Hg). We divided the patients into phenogroups based upon previous work showing different systolic and diastolic phenotypes and their combination [12]. As that work preceded the inclusion of biomarkers as a marker of SBHF, we added a 4th group characterized by abnormal biomarkers alone.

### Cardiopulmonary exercise testing

As described in the Methods paper [14], maximal cardiopulmonary exercise tests (CPET) were obtained using bicycle stress, with standard ECG monitoring and expired gas analysis. We assessed the duration of exercise, peak VO_2_, and peak respiratory exchange rate (RER; >1.05 indicates adequate test performance).

### Statistical analysis

For the baseline analysis, we considered all candidates undergoing echocardiography (including those ultimately excluded owing to normal exercise capacity). The distribution and characteristics of patients with preserved and reduced functional capacity (based on the peak VO_2_ cut-point of < 75% of predicted normal) were compared. To elucidate the differences between the identified phenogroups, we used the chi-square test to assess the significance of differences between the clustering groups for categorical variables. The Wilcoxon rank-sum test was utilized to compare the medians between groups for non-normally distributed continuous variables and t-test for continuous variables that followed a normal distribution. The association of baseline variables with key components of the phenogroups were assessed in multivariable linear stepwise models, adjusted for demographics (age, sex, race), clinical features and biomarkers (blood pressure, NT-proBNP, hs-cTnT, Hb A1c, eGFR), echocardiographic findings (LV function, geometry and diastolic markers), and exercise testing (peak VO_2,_ exercise duration, peak RER). The strength of association between clinical features and the phenogroups were assessed using a stepwise model selection approach for the multivariable logistic models, with odds ratios (OR) and 95% confidence intervals (CI) reported. Missing variables were addressed through imputation using the MICE algorithm.

All statistical analyses were performed using the R version 4.2.3 (R Foundation for Statistical Computing, Vienna, Austria. URL: https://www.R-project.org/). P values are two-sided with values < 0.05 considered statistically significant. The datasets generated during and/or analyzed during the current study are not publicly available due to the ongoing ARISE-HF Trial. 

## Results

### Patient selection

**Fig. 1:**
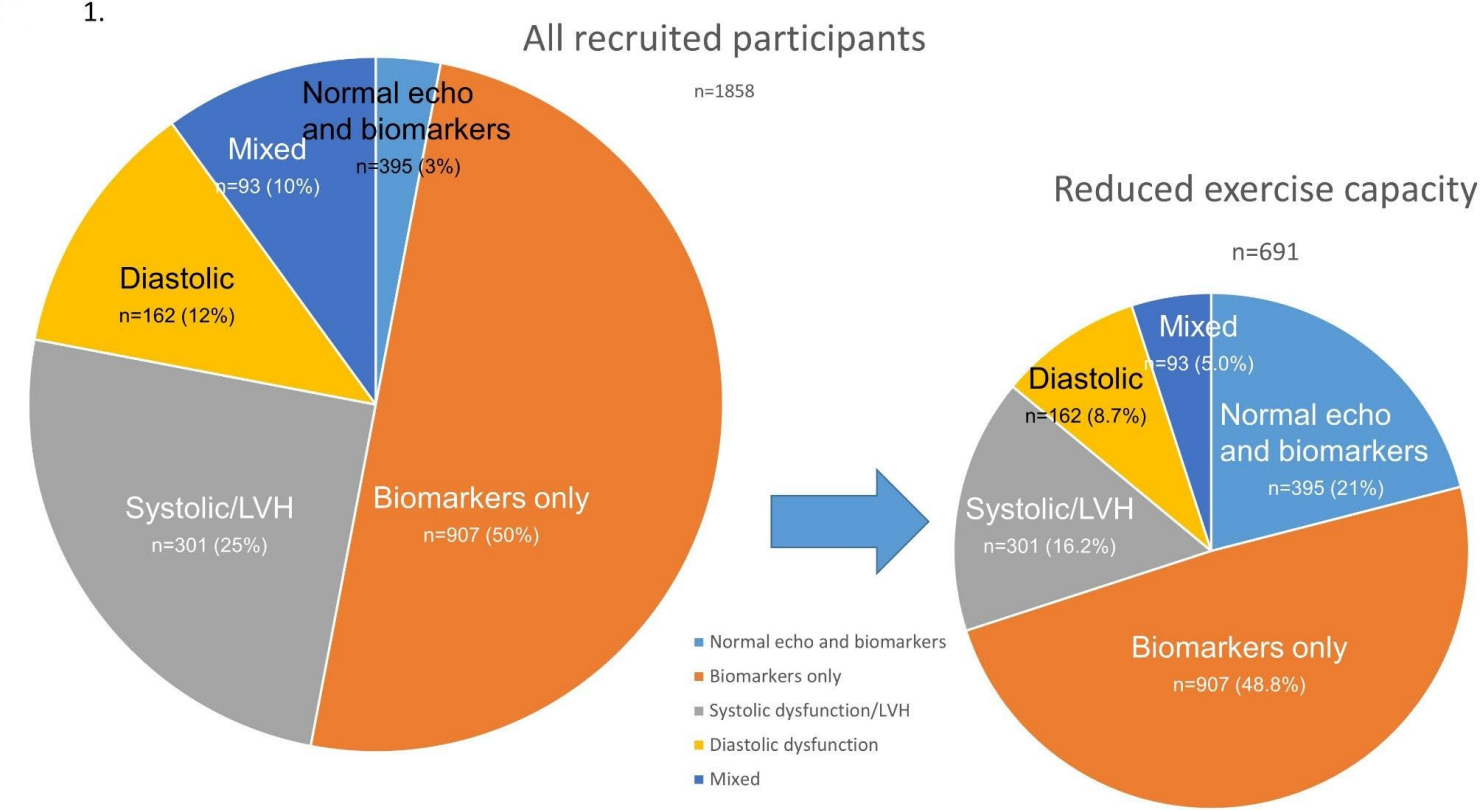
Selection of patients with DbMD and impaired functional capacity, and follow-up

The recruitment, selection and follow-up of patients is summarized in Fig. 1. Clinical and echocardiographic evaluation was undertaken in 1858 individuals *≥* 60 years old with T2DM (or *≥* 40 with > 10 years of T2DM or renal impairment) who had no history or symptoms of CVD (Table 1). Of these, 395 had normal echocardiographic and biomarker results and were excluded; this resulted in a baseline DbMD cohort of 1463 participants. This overall group with DbMD (age 67 years, 50% women) was ethnically diverse and represented patients with T2DM, at risk of HF (average T2DM duration of 14 years). Overall, a quarter had an NT-proBNP *≥* 125 ng/L and hs-cTnT was at or above the 99th percentile for a healthy population in 20% of the participants. While most study participants had a history of hypertension, by study protocol, only those well-controlled hypertension– most commonly taking angiotensin converting enzyme inhibitors or angiotensin II receptor blockers—were enrolled. Again, by study protocol, recruitment was restricted to patients with excellent glycemic control, with therapy most commonly including metformin, and with significant numbers of patients with SGLT2 inhibitors and GLP-1 receptor agonists.

**Table 1:** Baseline characteristics of 1463 DbMD patients in the four phenotype groups

	Full cohort	Elevated Biomarkers	Systolic/LVH	Diastolic	Overlap	*p*
n	1463	907 (62%)	301 (21%)	162 (11%)	93 (6%)	
*Demographic and clinical features*						
Age (mean (SD))	67.3 (7.5)	67.3 (7.5)	66.0 (7.4)	68.5 (8.0)	69.6 (6.9)	< 0.001
Male sex (%)	797 (54.5)	496 (54.7)	178 (59.1)	90 (55.6)	33 (35.5)	0.001
Race (%) - White	925 (63.2)	597 (65.8)	175 (58.1)	105 (64.8)	48 (51.6)	0.008
Hispanic	300 (20.5)	179 (19.7)	58 (19.3)	36 (22.2)	27 (29.0)	
Black	116 (7.9)	61 (6.7)	39 (13.0)	10 (6.2)	6 (6.5)	
Asian	99 (6.8)	58 (6.4)	23 (7.6)	10 (6.2)	8 (8.6)	
AI/AN	5 (0.3)	3 (0.3)	2 (0.7)	0 (0.0)	0 (0.0)	
Other	18 (1.2)	9 (1.0)	4 (1.3)	1 (0.6)	4 (4.3)	
SBP (mmHg, mean [SD])	130 (13)	130 (13)	128 (12)	133 (13)	131 (17)	0.008
DBP (mmHg, mean [SD])	75.99 (8.83)	76.08 (8.87)	76.18 (8.63)	76.19 (8.27)	74.63 (10.00)	0.56
*Laboratory Test*						
NT-proBNP (ng/L, median [IQR])	77 [39, 139]	77 [44, 138]	62 [29, 118]	93 [45, 161]	118 [56, 200]	< 0.001
hs-cTnt (ng/L, median [IQR])	9 [7, 14]	9 [7, 13]	9 [6, 13]	11 [8, 15]	9 [6, 14]	0.02
HbA1 (%, mean [SD])	7.18 (1.16)	7.22 (1.27)	7.20 (1.01)	7.05 (0.83)	6.94 (0.91)	0.06
Hgb (g/dL, mean [SD])	13.68 (1.47)	13.70 (1.50)	13.76 (1.48)	13.62 (1.34)	13.28 (1.40)	0.05
eGFR (mL/min/1.73 m2, mean [SD])	78.31 (18.06)	77.7 (18.4)	80.7 (17.7)	78.9 (16.5)	75.2 (18.1)	0.03
*Echocardiogram*						
LVEF (%, median [IQR])	62 [59, 65]	63 [60, 66]	60 [57, 63]	63 [60, 66]	62 [60, 65]	< 0.001
GLS (%, median [IQR])	-17.4 [-19.4, -15.1]	-18.8 [-20.2, -17.9]	-14.8 [-15.5, -13.9]	-19.1 [-20.9, -17.7]	-15.4 [-18.9, -14.1]	< 0.001
LAVI (ml/m^2^, median [IQR])	23 [19, 29]	22 [19, 26]	22 [18, 26]	34 [25, 37]	35 [26, 37]	< 0.001
LVMI (g/m^2^, median [IQR])	73 [62, 88]	68 [60, 79]	74 [63, 91]	88 [67, 94.30]	101.50 [95.75, 115]	< 0.001
E/e’ (median [IQR])	9.3 [7.7, 11.5]	8.7 [7.4, 10.3]	8.9 [7.4, 10.2]	13.7 [10.5, 14.8]	14.7 [13.2, 16.5]	< 0.001
RVSP (mmHg, median [IQR])	23 [19, 28]	23 [19, 26]	22 [18, 26]	28 [22, 36]	27 [21, 32]	< 0.001
*CPET*						
PEAK VO2 (mL/kg/min, mean [SD])	16.26 (4.84)	16.52 (4.91)	16.35 (4.58)	16.38 (5.27)	14.34 (3.99)	0.003
Duration of CPET (min, mean ± SD)	9.88 (2.86)	9.98 (2.79)	10.08 (2.94)	9.80 (3.07)	8.97 (2.49)	0.02
Peak RER (mean ± SD)	1.16 (0.10)	1.16 (0.10)	1.17 (0.11)	1.15 (0.11)	1.16 (0.10)	0.22

AI/AN - American Indian/Alaskan native, RER = respiratory exchange rate

### Phenogroups of T2DM at baseline

Most participants had abnormal biomarkers alone (Fig. 1; Table 1); this group had more female participants and a shorter T2DM duration than those with the echocardiographic phenotypes. The biomarker group had a slightly higher EF (although both groups were in the normal range), and mean functional capacity, sedentariness and symptom status were similar (Appendix Table 1). Although the number with abnormal biomarkers fell when an NT-proBNP cut-off of 125 ng/L was used, the overall comparison with the group with abnormal echocardiograms was similar (Appendix Table 2).

**Table 2:** Baseline characteristics of 1463 patients in the ARISE HF Trial

	Preserved exercise	Reduced exercise	*p*
n	794	669	
Age (mean (SD))	67.14 (7.82)	67.46 (7.14)	0.43
Male sex (%)	460 (57.9)	337 (50.4)	0.005
Race (%)			0.15
White	501 (63.1)	424 (63.4)	
Hispanic	154 (19.4)	146 (21.8)	
Black	75 (9.4)	41 (6.1)	
Asian	50 (6.3)	49 (7.3)	
AI/AN	2 (0.3)	3 (0.4)	
Other	12 (1.5)	6 (0.9)	
SBP (mmHg, mean [SD])	132.94 (18.04)	129.17 (11.22)	< 0.001
DBP (mmHg, mean [SD])	76.46 (10.32)	75.84 (8.29)	0.37
NT-proBNP (ng/L, median [IQR])	80.00 [42.00, 142.00]	73.00 [37.00, 136.00]	0.18
hs-cTnt (ng/L, median [IQR])	10.00 [7.00, 15.00]	9.00 [6.00, 12.00]	< 0.001
HbA1 (%, mean [SD])	7.35 (1.38)	6.98 (0.79)	< 0.001
Hgb (g/dL, mean [SD])	13.69 (1.54)	13.66 (1.40)	0.69
eGFR (mL/min/1.73 m2, mean [SD])	76.72 (19.40)	80.20 (16.15)	< 0.001
LVEF (%, median [IQR])	62.00 [58.00, 65.00]	62.00 [59.00, 66.00]	0.10
GLS (%, median [IQR])	-17.10 [-19.10, -14.90]	-17.70 [-19.50, -15.10]	0.10
LAVI (ml/m^2^, median [IQR])	22.90 [18.90, 27.80]	23.50 [19.10, 29.00]	0.12
LVMI (g/m^2^, median [IQR])	72.00 [61.00, 88.00]	74.00 [64.00, 88.30]	0.05
E/e’ (median [IQR])	9.10 [7.50, 11.20]	9.40 [7.80, 11.90]	0.002
RVSP (mmHg, median [IQR])	23.15 [19.00, 27.50]	23.40 [19.02, 27.67]	0.77
Peak VO2 (mL/kg/min, mean [SD])	17.99 (6.82)	15.68 (3.80)	< 0.001
Duration of CPET (min, mean ± SD)	10.28 (3.87)	9.75 (2.41)	0.02
Peak RER (mean ± SD)	1.11 (0.11)	1.18 (0.10)	< 0.001

The patients are divided into those with preserved (“screen failed”) and reduced exercise capacity

**Fig. 2:**
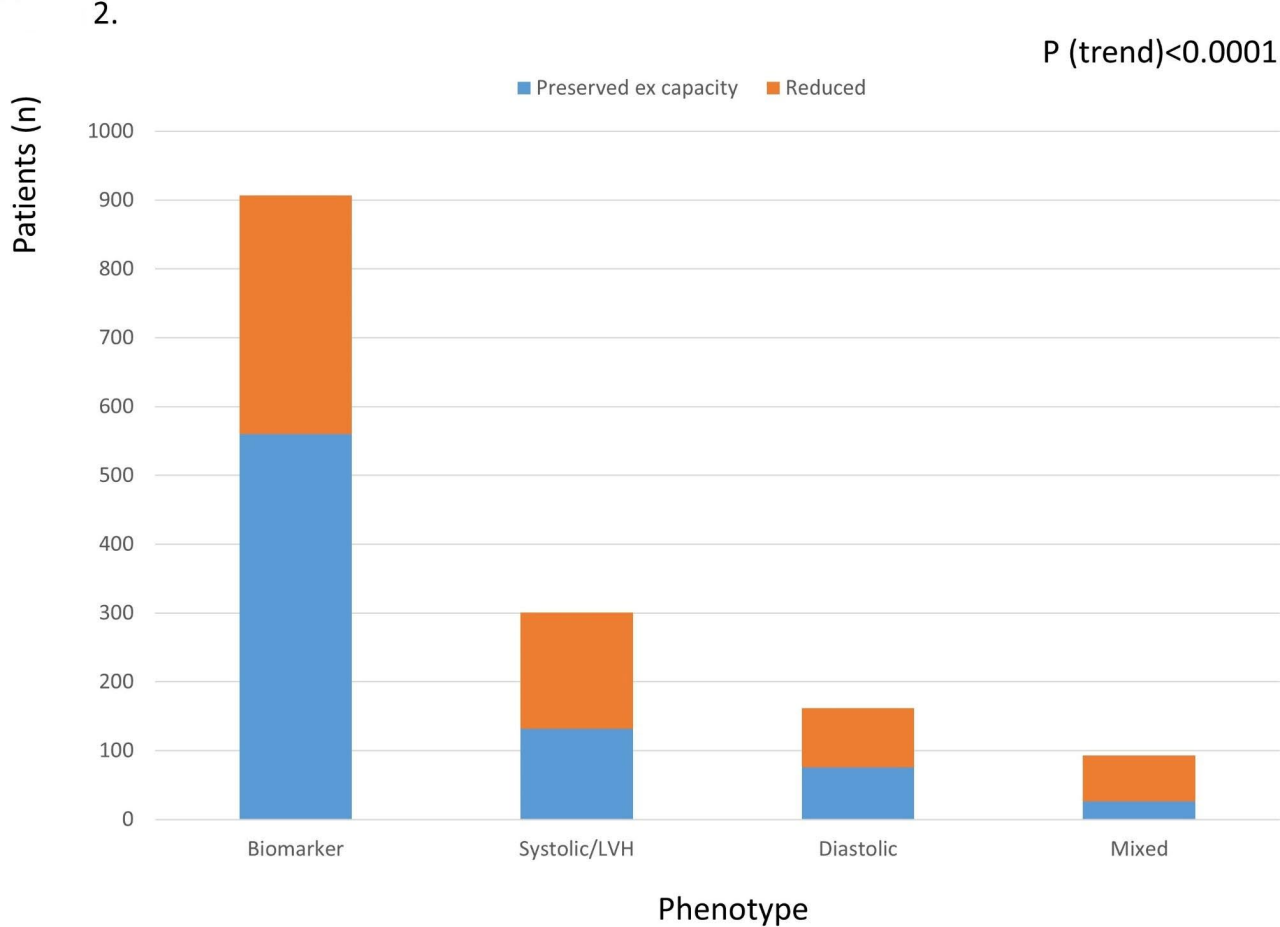
Proportion of participants with preserved and reduced exercise capacity according to baseline phenogroup. Reduced exercise capacity was most frequent in the mixed phenogroup

Of participants with an abnormal echocardiogram, the group with systolic dysfunction or LV hypertrophy had lower body-mass index than those with DD, and had significantly lower natriuretic peptide levels with greater exercise capacity. The 255 patients with DD either in isolation or combined with systolic dysfunction were the most distinct from the other groups (Appendix Table 3). These patients were older, more likely to be female and from racial/ethnic minority groups, with the highest average blood pressure (BP) and NT pro-BNP, and the most cardiac dysfunction and LV hypertrophy. The mixed group had the greatest proportion with impaired functional capacity (Fig. 2) (Table 2).

**Table 3:** Association of variables with the phenotypic cluster

	Elevated Biomarkers		Systolic/LVH		Diastolic		Overlap	
	Odds Ratio (95% CI)	p	Odds Ratio (95% CI)	p	Odds Ratio (95% CI)	p	Odds Ratio (95% CI)	p
Male sex							0.25 (0.12–0.51)	< 0.001
SBP (mmHg, mean [SD])							1.03 (1.00-1.05)	0.05
DBP (mmHg, mean [SD])	1.03 (1.01–1.04)	0.003	0.98 (0.96-1.00)	0.08			0.95 (0.91-1.00)	0.03
NT-proBNP (ng/L, median [IQR])*	1.31 (1.13–1.51)	< 0.001	0.60 (0.49–0.73)	< 0.001				
hs-cTnt (ng/L, median [IQR])*	1.33 (1.04–1.70)	0.02	0.66 (0.48–0.92)	0.01	1.54 (1.05–2.27)	0.03	0.59 (0.34–1.02)	0.05
LVEF (%, median [IQR])	0.97 (0.94-1.00)	0.08	1.04 (1.00-1.07)	0.06	0.95 (0.91-1.00)	0.04	1.07 (1.01–1.14)	0.03
GLS (%, median [IQR])	0.52 (0.48–0.57)	< 0.001	3.39 (2.90–3.96)	< 0.001	0.69 (0.62–0.76)	< 0.001	1.61 (1.41–1.85)	< 0.001
LAVI (ml/m^2^, median [IQR])	0.92 (0.89–0.94)	< 0.001	0.92 (0.88–0.95)	< 0.001	1.20 (1.16–1.25)	< 0.001	1.14 (1.09–1.19)	< 0.001
LVMI (g/m^2^, median [IQR])	0.97 (0.96–0.98)	< 0.001	1.05 (1.04–1.07)	< 0.001	0.95 (0.94–0.97)	< 0.001	1.06 (1.04–1.08)	< 0.001
E/e’ (median [IQR])	0.76 (0.72–0.82)	< 0.001	0.69 (0.63–0.76)	< 0.001	1.39 (1.29–1.50)	< 0.001	1.36 (1.23–1.51)	< 0.001
RVSP (mmHg, median [IQR])	0.93 (0.91–0.95)	< 0.001	0.94 (0.91–0.97)	< 0.001	1.17 (1.13–1.21)	< 0.001	1.07 (1.02–1.12)	0.004
Peak VO2 (mL/kg/min, mean [SD])	1.03 (1.00-1.06-)	0.07	0.97 (0.93–1.01)	0.15	1.06 (1.01–1.11)	0.02		

Age, race, GFR, Hb, HbA1C, exercise duration and RER were not associated *NT-proBNP and hs-cTnT are log-transformed

### Baseline features associated with phenotypic groups

**Fig. 3.**
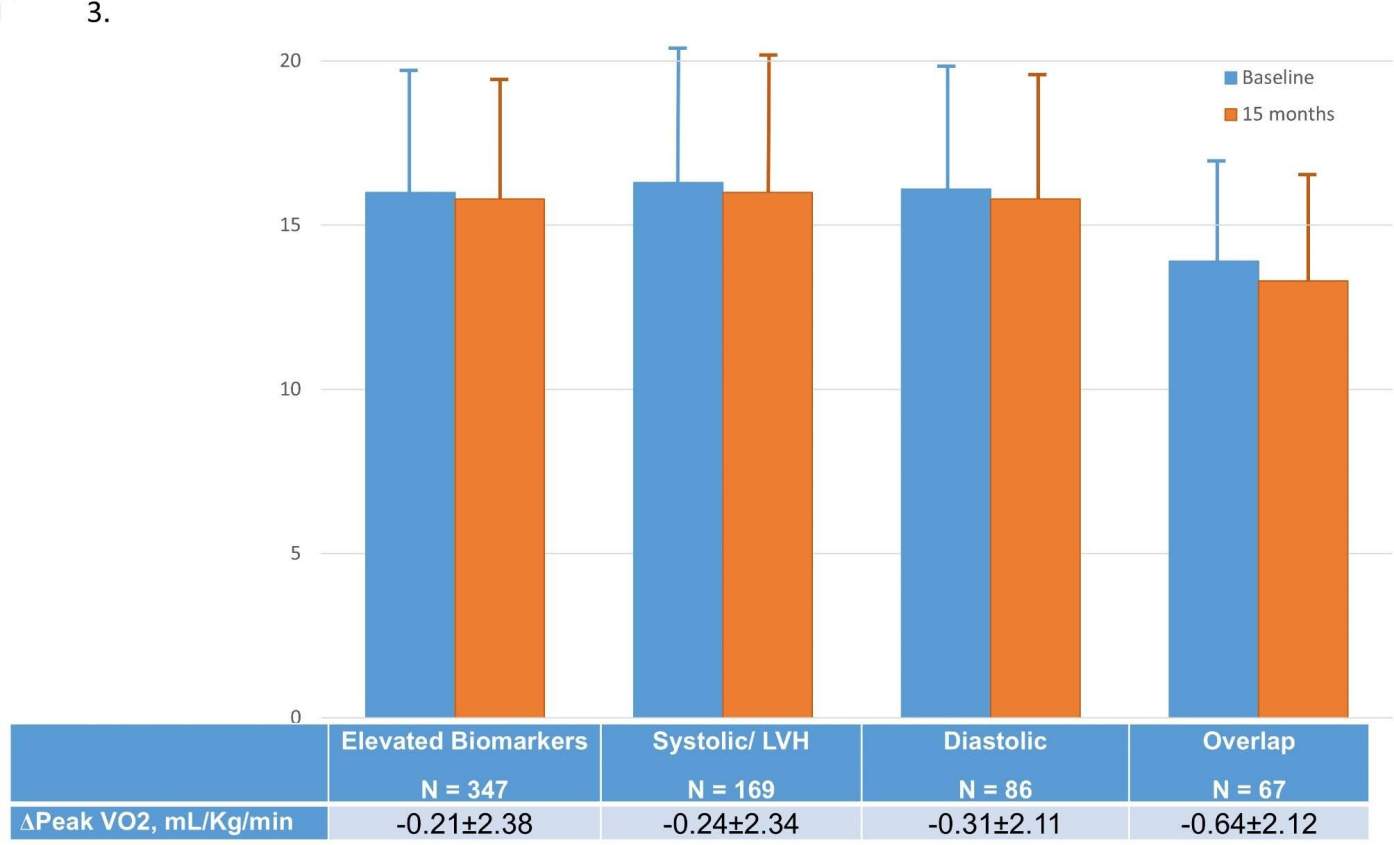
**Baseline, 15-month and change in peak VO**_**2**_** in each phenogroup**. While the 15-month VO_2_ was significantly lower (p = 0.001) in the overlap than the other groups, the changes were similar in each phenogroup

The association of clinical, echocardiographic and exercise variables with the phenogroups and their underlying physiologic variables are summarized in Tables 3 and 4, respectively. Age and male sex were associated with LVMI, diastolic parameters and NT-proBNP, but they were not strongly associated with phenogroups. However, there were significant associations between race and hsTnT. Although blood pressure was independently associated with GLS, E/e’ and NT-pro-BNP, systolic BP was only associated with the overlap group. Exercise capacity was independently associated with GLS and diastolic markers, and was only associated with the diastolic phenogroup (Fig. 3).

**Table 4:** Association of clinical, echocardiographic and exercise variables with key components of the phenotypes

	GLS		LVMI		E/e’		LAVI		NTproBNP		hs-cTnt	
	β	p	β	p	β	p	β	p	β	p	β	p
Age	0.011	0.25	-0.176	0.004	0.035	0.001			0.015	< 0.001		
Male sex			3.852	< 0.001	-0.812	< 0.001	0.843	0.01	-0.298	< 0.001	0.491	< 0.001
Hispanic^											-0.071	0.05
Black^											0.138	0.007
Asian^											-0.065	0.24
AI/AN^											-0.608	0.009
Other^											0.112	0.37
SBP (mmHg, mean [(SD]))	-0.014	0.004			0.013	0.04			0.011	< 0.001		
DBP (mmHg, mean [SD]mean (SD))					-0.022	0.03			-0.013	< 0.001		
NT-proBNP (ng/L, median [IQR])*			-0.636	0.15	-0.147	0.05	0.738	< 0.001			0.091	< 0.001
hs-cTnt (ng/L, median [IQR])*			1.719	0.03					0.265	< 0.001		
HbA1 (%, mean [(SD]))	0.046	0.34	-0.594	0.08	0.103	0.08			-0.116	< 0.001		
eGFR (mL/min/1.73 m2, mean [SD])							0.026	0.005	-0.009	< 0.001	-0.009	< 0.001
LVEF (%, median [IQR])	-0.211	< 0.001							-0.015	0.001		
GLS (%, median [IQR])			0.596	< 0.001			-0.241	< 0.001				
LAVI (ml/m^2^, median [IQR])	-0.038	< 0.001	0.958	< 0.001	0.131	< 0.001			0.016	< 0.001		
LVMI (g/m^2^, median [IQR])	0.015	< 0.001			0.052	< 0.001	0.127	< 0.001	-0.020	0.02	0.002	< 0.001
E/e’ (median [IQR])			1.795	< 0.001			0.626	< 0.001	0.012	< 0.001		
RVSP (mmHg, median [IQR])					0.040	< 0.001					0.003	0.07
PEAK VO2 (mL/kg/min, mean [SD])	-0.045	0.02			-0.094	< 0.001	0.126	0.008				
Duration of CPET (min, mean ± SD)	0.039	0.27			0.061	0.12	-0.172	0.04	-0.039	< 0.001	-0.021	< 0.001
Peak RER (mean ± SD)											-0.570	< 0.001

^Associations for race are referenced to white participants. *NT-proBNP and hsTnT are log-transformed

### Impaired baseline functional capacity

**Fig. 4:**
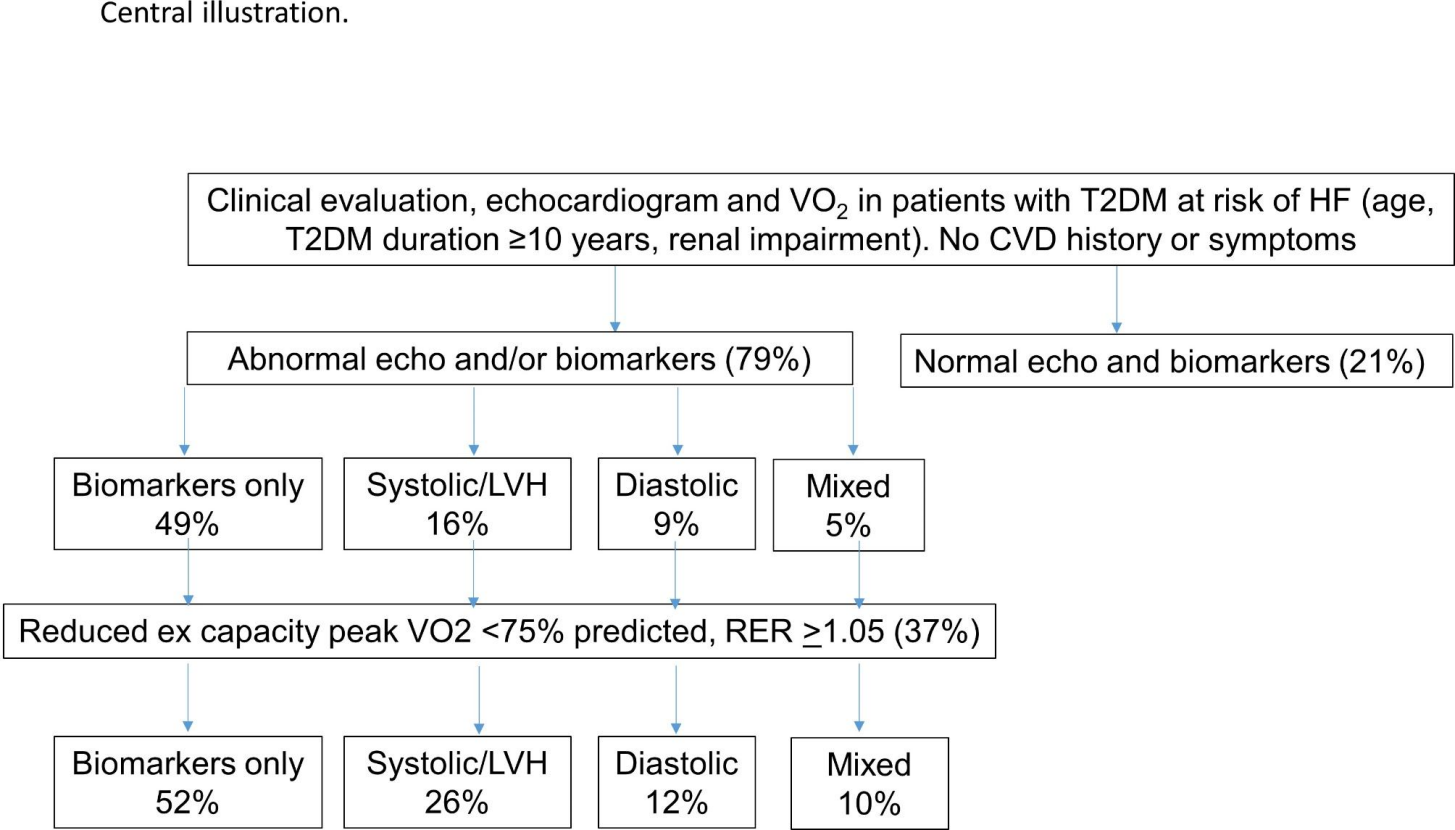
Central illustration: Distribution of abnormal biomarkers and echocardiograms in 1463 patients with diabetes-related myocardial dysfunction (DbMD) who had no history or symptoms of CVD, and in the subgroup with reduced functional capacity. Impaired exercise capacity was more common in those with abnormal echocardiography (especially with multiple abnormalities) and less frequent in those with isolated abnormal biomarkers (p < 0.0001)

The limited reporting of symptoms typically associated with overt HF was confirmed by the KCCQ scores (Table 1). The selection process identified reduced functional capacity, evidenced by CPET results, and confirmed by PASE scores, in fewer than half of the screened patients. Nonetheless, substantial reduction of exercise capacity (to < 75% predicted VO_2_) required for entry into the ARISE-HF trial was more commonly seen in the echocardiographic phenotypes (Fig. 4) and among older individuals (Table 2) even in those with near-normal KCCQ or PASE scores. Patients with preserved exercise capacity (excluded from the trial) were more frequent in the abnormal biomarker group (62%), and less frequent in systolic and diastolic dysfunction and mixed groups (47 to 28%, *p* < 0.0001) (Fig. 4). Appendix Table 4 shows the similarities of patients with impaired and preserved exercise capacity within each of the phenogroups.

### Follow-up functional capacity

The evolution of functional capacity according to phenogroup of patients with DbMD and reduced baseline functional capacity is shown in Appendix Table 5. Although functional capacity remained worst in the overlap group, the changes in functional capacity was similar (Fig. 3), as were changes in PASE and KCCQ scores.

## Discussion

In this study of HF risk evaluation, abnormal echocardiograms and/or biomarkers were identified in 79% of > 1800 patients with T2DM with heightened risk of HF despite a lack of manifest ischemic heart disease and with adequate glycemic control and blood pressure. The phenotypic presentation of this group with DbMD was heterogeneous, with the most common being abnormal biomarkers in the absence of echocardiographic abnormalities, followed by DD (alone or with systolic dysfunction), and a smaller group of isolated systolic dysfunction and/or LV hypertrophy. Reduced functional capacity was present in 46%, but this was also heterogeneous between groups - only 38% of patients with abnormal biomarkers, but > 50% of those with isolated and > 70% of those with multiple echocardiographic abnormalities. These findings suggest that a range of biological processes underlie DbMD. The follow-up data show that the evolution of functional capacity was similar, perhaps because patients were selected on the basis of impaired baseline functional capacity.

### Significance of subclinical dysfunction

Waiting for symptoms to develop before treating a patient with HF carries the risk that the underlying cardiac pathology may be too advanced to make a substantive response to disease-modifying therapy. The asymptomatic nature of early-stage HF is the basis of the recent American Diabetes Association recommendation to screen for HF in DM [2], so as to initiate cardioprotective treatments to prevent progression to overt HF. However, the potential disadvantage of a HF screening strategy is that effort and cost may be directed towards patients at low risk. Patients with DM are a readily-defined “at risk” group for HF [19], and may constitute an appropriate group for testing the concept of early recognition and treatment of Stage B HF [3]. These results indicate that selection of patients with T2DM at risk of HF, based on age, DM duration or renal impairment provides a very high frequency of abnormal studies (79%), very similar to the 81% prevalence of SBHF in the ARIC study HF [20]. The findings of most functional compromise in patients with both abnormal echo and biomarkers in this study also matched the outcomes in a 7-year follow-up of ARIC, which showed that SBHF defined by echocardiography (7%) or biomarkers (8.5%) were modestly associated with increased HF risk, with patients with both signals having the highest risk for HF (23%) [20]. Both results point towards a high prevalence of SBHF with the application of current definitions– many of these patients will not progress to HF. Using a combination of multiple physiologic parameters could identify a manageable subgroup at risk for progression to overt HF.

The definition of DbMD is variable. From a mechanistic standpoint, DbMD is associated with oxidative stress, vascular inflammation and endothelial dysfunction [21]. These phenomena may be evidenced by changes in myocardial mechanics (hence, abnormal GLS), and release of natriuretic peptides. Several recent studies have shown gradations of prevalence, prognosis and potential therapeutic response with categories based on abnormal biomarkers and/or echocardiography [22], different degrees of echocardiographic disturbance [23], and their combination [24]. This study adds to these observations by showing gradations in functional disturbance using the same approach. While the cut-points for all of these parameters is arbitrary, it was reassuring to find that the biomarker and echocardiographic groups showed similar differences at the two selected cut-offs.

### Impaired functional capacity

The underlying mechanisms of heart muscle disease in our study participants may also contribute to impaired functional capacity (evidenced in this study by lower PASE score and VO_2_)– a common finding in SBHF [25] despite the asymptomatic state of these patients (evidenced here by minimal abnormalities of KCCQ). While echocardiographic [13, 24], biomarker [26] and exercise data [27] have prognostic significance in this setting, none of these associations are specific for DbMD. Reduced VO_2_ in particular may be driven by DM-related pathology throughout the oxygen transport system, including pulmonary, cardiovascular and skeletal muscle complications [25]. Nonetheless, the point remains that none are exclusively found in early DbMD– implying that a screening process may need to include multiple physiologic modalities.

Among those considered in this analysis, 691 with impaired functional capacity (arguably a more advanced population with DbMD) were randomized to receive AT-001, a highly-potent aldose reductase inhibitor. Interestingly, the frequency of lower functional capacity varied according to the phenotype of DbMD. However, aldose reductase therapy did not stabilize exercise capacity– either in the group overall or in the phenotypic subgroups. These remained stable over follow-up, there was no difference in the evolution of exercise impairment among those with reduced exercise capacity at baseline.

### Strengths and limitations

This study provides detailed phenotyping of a large number of patients with T2DM at risk of HF, but with good glycemic and BP control. The results are obtained from the baseline assessment of a clinical trial, and this provides the benefit of uniform acquisition and interpretation provided by core-laboratories for echocardiography and cardiopulmonary testing. In clinical practice, the phenotypes could be modified by less adequate glycemic and BP control, and co-existent chronic kidney disease and other comorbidities, whereas a common feature of trials is the selection of patients with controlled glycemia and blood pressure. On the other hand, the control of these aspects does enable the analysis to evaluate the underlying myocardial consequences of DbMD, rather the acute responses of the myocardium to metabolic disturbance. The lack of association of the phenogroups with progression of exercise may reflect the level of baseline impairment in all participants undergoing follow-up; only participants with impaired exercise capacity were followed up after 12 months.

### Conclusions

These results from the ARISE-HF trial show a high prevalence of SBHF, as well as heterogeneity of echocardiography, biomarkers and exercise capacity in early DbMD. However, a small group of patients with multiple underlying pathophysiologies have the most impaired functional capacity.

## Electronic supplementary material

Below is the link to the electronic supplementary material.

Supplementary Material 1

## Data Availability

No datasets were generated or analysed during the current study.
