# Supplementary material for: Echocardiographic phenotypes of diabetic myocardial disorder: evolution over 15 months follow-up in the ARISE-HF trial
Source: Cardiovasc Diabetol. 2025 Jan 13;24:16. doi: 10.1186/s12933-024-02554-y (PMC11730511; doi:10.1186/s12933-024-02554-y)
Supplement: Supplementary file 1 — Supplementary Material 1 [file 12933_2024_2554_MOESM1_ESM.docx]

**Appendix Table 1. Comparison of biomarker group and rest of patients in ARISE-HF.**

|  |  | Elevated Biomarker (907) | Echo abnormalities (556) | p |
| --- | --- | --- | --- | --- |
| **Demographic/clinical variables** | Age (mean (SD)) | 67.25 (7.48) | 67.33 (7.59) | 0.85 |
|  | Male (%) | 496 (54.7) | 301 (54.1) | 0.88 |
|  | Race (%) |  |  | 0.10 |
|  | White | 597 (65.8) | 328 (59.0) |  |
|  | Hispanic | 179 (19.7) | 121 (21.8) |  |
|  | Black | 61 (6.7) | 55 (9.9) |  |
|  | Asian | 58 (6.4) | 41 (7.4) |  |
|  | American Indian or Alaskan Native | 3 (0.3) | 2 (0.4) |  |
|  | Other | 9 (1.0) | 9 (1.6) |  |
|  | SBP (mean (SD)) | 130.31 (13.26) | 129.87 (13.38) | 0.62 |
|  | DBP (mean (SD)) | 76.08 (8.87) | 75.90 (8.79) | 0.76 |
| **Laboratory Tests** | NT-proBNP (median [IQR]) | 77.00 [44.00, 138.00] | 78.00 [34.00, 142.00] | 0.68 |
|  | hs-cTnt (median [IQR]) | 9.00 [7.00, 13.00] | 9.00 [7.00, 14.00] | 0.79 |
|  | HBA1 (mean (SD)) | 7.22 (1.27) | 7.11 (0.95) | 0.07 |
|  | Hgb (mean (SD)) | 13.70 (1.50) | 13.64 (1.43) | 0.49 |
|  | eGFR (mean (SD)) | 77.72 (18.38) | 79.27 (17.50) | 0.11 |
| **Echocardiogram** | LVEF (median [IQR]) | 63.00 [60.00, 66.00] | 61.00 [58.00, 64.00] | <0.001 |
|  | GLS (median [IQR]) | -18.80 [-20.20, -17.90] | -15.20 [-17.00, -14.20] | <0.001 |
|  | LAVI (median [IQR]) | 22.00 [18.60, 26.00] | 25.10 [20.00, 34.10] | <0.001 |
|  | LVMI (median [IQR]) | 68.00 [60.00, 79.00] | 83.00 [67.00, 98.00] | <0.001 |
|  | EE (median [IQR]) | 8.70 [7.38, 10.30] | 10.30 [8.40, 13.90] | <0.001 |
|  | RVSP (median [IQR]) | 22.60 [18.80, 26.00] | 24.05 [19.52, 30.60] | <0.001 |
| **CPET** | PEAK VO2 (mean (SD)) | 16.52 (4.91) | 15.99 (4.77) | 0.10 |
|  | Duration of CPET test, min, mean ± SD | 9.98 (2.79) | 9.79 (2.93) | 0.32 |
|  | Peak respiratory exchange rate, mean ± SD | 1.16 (0.10) | 1.16 (0.11) | 0.84 |
| **Questionnaires** | KCCQ physical limitation score, mean ± SD |  |  |  |
|  | KCCQ symptom stability score, mean ± SD |  |  |  |
|  | KCCQ symptom burden score, mean ± SD |  |  |  |
|  | KCCQ self-efficacy score, mean ± SD |  |  |  |
|  | KCCQ quality of life score, mean ± SD |  |  |  |
|  | KCCQ social limitation score, mean ± SD |  |  |  |
|  | KCCQ total symptom score, mean ± SD |  |  |  |
|  | KCCQ clinical summary score, mean ± SD |  |  |  |
|  | KCCQ overall summary score, mean ± SD |  |  |  |
|  | PASE score, mean ± SD |  |  |  |

**Appendix Table 2. Comparison of biomarkers group with an NT-proBNP cut-off of 125 ng/L and the rest of the participants.**

|  | Elevated Biomarkers | All | p |
| --- | --- | --- | --- |
| n | 751 | 556 |  |
| AGE (mean (SD)) | 67.46 (7.49) | 67.33 (7.59) | 0.76 |
| MALE (%) | 441 (58.7) | 301 (54.1) | 0.11 |
| RACE (%) |  |  | 0.08 |
| White | 500 (66.6) | 328 (59.0) |  |
| Hispanic | 148 (19.7) | 121 (21.8) |  |
| Black | 53 (7.1) | 55 (9.9) |  |
| Asian | 38 (5.1) | 41 (7.4) |  |
| American Indian or Alaskan Native | 3 (0.4) | 2 (0.4) |  |
| Other | 9 (1.2) | 9 (1.6) |  |
| SBP (mean (SD)) | 131 (14) | 130 (13) | 0.40 |
| DBP (mean (SD)) | 76 (9) | 76 (9) | 0.66 |
| **Laboratory Test** |  |  |  |
| NT-proBNP (median [IQR]) | 80.00 [35.00, 148.00] | 78.00 [34.00, 142.00] | 0.55 |
| hs-cTnt (median [IQR]) | 10.00 [7.00, 14.00] | 9.00 [7.00, 14.00] | 0.004 |
| HBA1 (mean (SD)) | 7.20 (1.25) | 7.11 (0.95) | 0.17 |
| Hgb (mean (SD)) | 13.73 (1.52) | 13.64 (1.43) | 0.26 |
| eGFR (mean (SD)) | 77.02 (18.62) | 79.27 (17.50) | 0.03 |
| **Echocardiodiogram** |  |  |  |
| LVEF (median [IQR]) | 63.00 [60.00, 66.00] | 61.00 [58.00, 64.00] | <0.001 |
| GLS (median [IQR]) | -18.90 [-20.40, -17.90] | -15.20 [-17.00, -14.20] | <0.001 |
| LAVI (median [IQR]) | 22.10 [18.60, 26.17] | 25.10 [20.00, 34.10] | <0.001 |
| LVMI (median [IQR]) | 69.00 [60.30, 80.00] | 83.00 [67.00, 98.00] | <0.001 |
| EE (median [IQR]) | 8.80 [7.40, 10.40] | 10.30 [8.40, 13.90] | <0.001 |
| RVSP (median [IQR]) | 22.60 [18.90, 26.00] | 24.05 [19.52, 30.60] | <0.001 |
| **CPET** |  |  |  |
| PEAKVO2 (mean (SD)) | 16.65 (5.08) | 15.99 (4.77) | 0.06 |
| Duration of CPET test, min, mean ± SD | 1.16 (0.10) | 1.16 (0.11) | 0.84 |
| Peak respiratory exchange rate, mean±SD | 16.65 (5.08) | 15.99 (4.77) | 0.06 |

**Appendix Table 3. Comparison of phenotype groups in the screened cohort.**

|  | Elevated Biomarkers of Heart disease | Systolic/LVH cluster | P (biomarkers vs systolic) | Diastolic + Overlap | P (biomarkers vs diastolic+overlap) | P (systolic vs diastolic+overlap) |
| --- | --- | --- | --- | --- | --- | --- |
| n | 907 | 301 |  | 255 |  |  |
| AGE (mean (SD)) | 67.25 (7.48) | 66.05 (7.36) | 0.02 | 68.91 (7.58) | 03 | <01 |
| MALE (%) | 496 (54.7) | 178 (59.1) | 0.20 | 123 (48.2) | 0.08 | 0.01 |
| Race (%) |  |  | 0.02 |  | 0.31 | 0.07 |
| White | 597 (65.8) | 175 (58.1) |  | 153 (60.0) |  |  |
| Hispanic | 179 (19.7) | 58 (19.3) |  | 63 (24.7) |  |  |
| Black | 61 (6.7) | 39 (13.0) |  | 16 (6.3) |  |  |
| Asian | 58 (6.4) | 23 (7.6) |  | 18 (7.1) |  |  |
| American Indian/Alaskan | 3 (0.3) | 2 (0.7) |  | 0 (0.0) |  |  |
| Other | 9 (1.0) | 4 (1.3) |  | 5 (2.0) |  |  |
| SBP (mean (SD)) | 130.31 (13.26) | 127.81 (12.18) | 0.02 | 131.96 (14.21) | 0.14 | 01 |
| DBP (mean (SD)) | 76.08 (8.87) | 76.18 (8.63) | 0.89 | 75.62 (8.96) | 0.53 | 0.51 |
| **Laboratory Tests**  NT-proBNP (median [IQR])  hs-cTnt (median [IQR])  HbA1c (%, mean (SD))  Hb (mean (SD))  eGFR (mean (SD)) | 77 [44, 138]  9 [7, 13]  7.22 (1.27)  13.70 (1.50)  77.72 (18.38) | 62 [29, 118]  9 [6, 13.25]  7.20 (1.01)  13.76 (1.48)  80.71 (17.66) | <.0001  0.13  0.75  0.51  0.01 | 101 [47, 173]  10 [7, 14]  7.01 (0.86)  13.50 (1.37)  77.58 (17.19) | <.0001  0.24  0.01  0.06  0.91 | <.0001  0.04  0.02  0.03  0.04 |
| **Echocardiogram**  LVEF (median [IQR])    GLS (median [IQR])    LAVI (median [IQR])    LVMI (median [IQR])    EE (median [IQR])    RVSP (median [IQR]) | 63 [60, 66]  -18.8 [-20.2, -17.9]  22 [19, 26]  68 [60, 79]  8.7 [7.4, 10.3]  22.6 [18.8, 26] | 60 [57, 63]  -14.8 [-15.5, -13.9]  22 [18, 26]  74 [63, 91]  8.9 [7.4, 10.2]  22.3 [18.2, 25.7] | <.0001  <.0001  0.26  <01  0.42  0.40 | 63 [60, 66]  -18.1 [-20, -15.5]  34 [25, 37]  92 [74, 102]  14 [11.5, 15.7]  27.9 [21.9, 35] | 0.45  <.0001  <.0001  <.0001  <.0001  <.0001 | <.0001  <.0001  <.0001  <.0001  <.0001  <.0001 |
| **CPET**  PEAK VO2 (mean (SD))  Duration of CPET test, min, mean ± SD  Pk RER, mean ± SD | 16.52 (4.91)  9.98 (2.79)  1.16 (0.10) | 16.35 (4.58)  10.08 (2.94)  1.17 (0.11) | 0.66  0.66  0.18 | 15.63 (4.93)  9.49 (2.89)  1.15 (0.11) | 0.03  0.04  0.32 | 0.12  0.04  0.05 |

**Appendix Table 4. Distribution of phenotypes in randomized (impaired exercise capacity) and screen-failure patients.** The randomized group had a greater proportion of patients with elevated biomarkers alone, and a smaller number of patients with a mixed phenotype (p<0.0001).

|  | **Elevated Biomarkers (907)** | | **Systolic/LVH (301)** | | **Diastolic (162)** | | **Overlap (93)** | |
| --- | --- | --- | --- | --- | --- | --- | --- | --- |
| n | Trial 347 (23.7%) | S/Fail 560 (38.3%) | Trial 169 (11.6%) | S/Fail 132 (9.0%) | Trial 86 (5.9%) | S/Fail 76  (5.2%) | Trial 67 (4.6%) | S/Fail 26 (1.8%) |
| Age (mean (SD)) | 67.70 (7.01) | 66.97 (7.75) | 66.88 (7.11) | 64.99 (7.56) | 66.27 (7.73) | 71.01 (7.56) | 69.06 (6.93) | 70.88 (6.62) |
| Male (%) | 168 (48.4) | 328 (58.6) | 96 (56.8) | 82 (62.1) | 52 (60.5) | 38 (50.0) | 21 (31.3) | 12 (46.2) |
| Race (%) |  |  |  |  |  |  |  |  |
| Black or African American | 19 (5.5) | 47 (8.4) | 19 (11.2) | 21 (15.9) | 6 (7.0) | 4 (5.3) | 4 (6.0) | 3 (11.5) |
| Other | 30 (8.6) | 146 (26.1) | 19 (11.2) | 34 (25.8) | 7 (8.1) | 24 (31.5) | 7 (10.4) | 14 (53.9) |
| White | 298 (85.9) | 367 (65.5) | 131 (77.5) | 77 (58.3) | 73 (84.9) | 48 (63.2) | 56 (83.6) | 9 (34.6) |
| BMI (mean (SD)) | 30 (4) | N/A | 30.33 (4.13) | N/A | 31.03 (4.87) | N/A | 31.88 (4.89) | N/A |
| SBP (mean (SD)) | 130 (12) | 131 (17) | 127 (11) | 130 (17) | 130(10) | 137 (15) | 128(12) | 148 (28) |
| **Medical History** |  |  |  |  |  |  |  |  |
| Duration of T2DM, yrs (mean (SD)) | 12.92 (10.13) | N/A | 14.91 (8.08) | N/A | 15.38 (10.82) | N/A | 16.20 (10.30) | N/A |
| Hypertension(%) | 259 (74.6) | N/A | 129 (76.3) | N/A | 67 (77.9) | N/A | 54 (80.6) | N/A |
| Dyslipidemia (%) | 52 (15.0) | N/A | 30 (17.8) | N/A | 17 (19.8) | N/A | 13 (19.4) | N/A |
| **Medications** |  |  |  |  |  |  |  |  |
| ACEi-ARBs (%) | 256 (73.8) | N/A | 120 (71.0) | N/A | 73 (84.9) | N/A | 53 (79.1) | N/A |
| B-Blockers (%) | 90 (25.9) | N/A | 30 (17.8) | N/A | 23 (26.7) | N/A | 17 (25.4) | N/A |
| MRA (%) | 9 (2.6) | N/A | 4 (2.4) | N/A | 2 (2.3) | N/A | 4 (6.0) | N/A |
| Hydrochlorothiazide (%) | 60 (17.3) | N/A | 32 (18.9) | N/A | 25 (29.1) | N/A | 11 (16.4) | N/A |
| SGLT2 (%) | 110 (31.7) | N/A | 54 (32.0) | N/A | 26 (30.2) | N/A | 20 (29.9) | N/A |
| GLP1 (%) | 78 (22.5) | N/A | 57 (33.7) | N/A | 20 (23.3) | N/A | 14 (20.9) | N/A |
| Statin(%) | 271 (78.1) | N/A | 148 (87.6) | N/A | 66 (76.7) | N/A | 50 (74.6) | N/A |
| Metformin(%) | 266 (76.7) | N/A | 129 (76.3) | N/A | 60 (69.8) | N/A | 45 (67.2) | N/A |
| Sulfonylureas (%) | 88 (25.4) | N/A | 30 (17.8) | N/A | 18 (20.9) | N/A | 16 (23.9) | N/A |
| Insulin (%) | 78 (22.5) | N/A | 49 (29.0) | N/A | 31 (36.0) | N/A | 18 (26.9) | N/A |
| DDP4 (%) | 40 (11.5) | N/A | 22 (13.0) | N/A | 5 (5.8) | N/A | 12 (17.9) | N/A |
| **Laboratory Test** |  |  |  |  |  |  |  |  |
| NT-proBNP (median [IQR]) | 77.00 [46.00, 141.50] | 77.00 [42.00, 132.00] | 61.00 [28.00, 117.50] | 64.00 [29.00, 122.25] | 70.00 [37.00, 125.25] | 107.00 [59.50, 189.00] | 98.00 [42.50, 161.00] | 184.00 [119.25, 358.50] |
| hs-cTnt (median [IQR]) | 9.00 [6.00, 12.00] | 10.00 [7.00, 14.00] | 8.00 [6.00, 12.00] | 9.00 [6.00, 15.00] | 11.00 [8.00, 13.75] | 11.50 [8.00, 16.00] | 8.00 [6.00, 10.00] | 13.00 [8.25, 19.75] |
| HbA1c (%, mean (SD)) | 6.95 (0.81) | 7.39 (1.47) | 7.02 (0.81) | 7.43 (1.19) | 7.11 (0.81) | 6.97 (0.85) | 6.84 (0.57) | 7.19 (1.46) |
| Hgb (mean (SD)) | 13.71 (1.37) | 13.69 (1.57) | 13.70 (1.49) | 13.85 (1.47) | 13.68 (1.34) | 13.55 (1.34) | 13.29 (1.32) | 13.26 (1.61) |
| eGFR (mean (SD)) | 79.67 (16.02) | 76.51 (19.63) | 81.85 (16.16) | 79.26 (19.39) | 81.10 (16.83) | 76.46 (15.93) | 77.63 (15.82) | 69.04 (22.27) |

**Appendix Table 5.** Evolution of peak VO2 and functional status over 15 month follow-up in participants treated with placebo.

| **Phenogroup (n)** | **Elevated Biomarkers**  **N = 117** | **Systolic/ LVH**  **N = 54** | p | **Diastolic**  **N = 28** | P | **Overlap**  **N = 24** | P |
| --- | --- | --- | --- | --- | --- | --- | --- |
| **Primary Endpoint** |  |  |  |  |  |  |  |
| Peak VO2, Month 15, mL/Kg/min, mean ± SD | 15.50 (3.92) | 15.40 (4.48) | 0.90 | 15.80 (4.28) | 0.75 | 12.32 (3.90) | **0.001** |
| Peak VO2, Change, mL/Kg/min, mean ± SD | -0.43 (2.25) | -0.43 (2.39) | 0.98 | 0.14 (2.24) | 0.31 | -0.90 (1.73) | 0.37 |
| **PASE Score, Month 15** |  |  |  |  |  |  |  |
| PASE score, Month 15, mean ± SD | 150.35 (81.79) | 163.48 (116.30) | 0.43 | 176.47 (81.48) | 0.19 | 135.81 (77.56) | 0.47 |
| PASE score, change, mean ± SD | -8.11 (86.64) | -7.38 (97.97) | 0.97 | 19.98 (73.50) | 0.19 | 11.99 (73.63) | 0.36 |
| **KCCQ Score, Month 15** |  |  |  |  |  |  |  |
| KCCQ physical limitation score, mean ± SD | 87.43 (17.95) | 84.01 (21.06) | 0.30 | 96.03 (7.62) | 0.03 | 78.92 (22.71) | 0.07 |
| KCCQ symptom stability score, mean ± SD | 51.49 (15.34) | 54.41 (11.94) | 0.24 | 52.38 (10.91) | 0.80 | 51.25 (9.85) | 0.95 |
| KCCQ symptom burden score, mean ± SD | 89.32 (17.24) | 88.89 (15.43) | 0.88 | 96.03 (6.78) | 0.08 | 85.83 (19.52) | 0.42 |
| KCCQ self-efficacy score, mean ± SD | 83.75 (25.34) | 78.92 (28.12) | 0.29 | 88.10 (25.15) | 0.48 | 91.88 (13.00) | 0.17 |
| KCCQ quality of life score, mean ± SD | 87.54 (19.28) | 83.82 (19.47) | 0.27 | 93.65 (9.83) | 0.16 | 84.17 (23.55) | 0.49 |
| KCCQ social limitation score, mean ± SD | 90.77 (18.43) | 86.48 (20.70) | 0.24 | 96.98 (6.32) | 0.14 | 84.72 (25.83) | 0.24 |
| KCCQ total symptom score, mean ± SD | 88.72 (17.24) | 89.26 (14.14) | 0.85 | 96.03 (5.63) | 0.06 | 84.53 (18.78) | 0.33 |
| KCCQ clinical summary score, mean ± SD | 88.07 (16.87) | 86.63 (16.06) | 0.62 | 96.03 (5.26) | 0.04 | 81.72 (18.86) | 0.13 |
| KCCQ overall summary score, mean ± SD | 88.79 (16.18) | 86.18 (16.18) | 0.35 | 95.68 (5.26) | 0.06 | 83.17 (20.22) | 0.18 |
| **KCCQ Score, Change month 0- 15** |  |  |  |  |  |  |  |
| KCCQ physical limitation score, mean ± SD | -0.73 (11.54) | -6.42 (19.36) | 0.03 | 0.79 (11.07) | 0.58 | -4.68 (11.80) | 0.19 |
| KCCQ symptom stability score, mean ± SD | -2.00 (16.55) | 2.45 (13.47) | 0.10 | -1.19 (16.73) | 0.84 | -2.50 (16.02) | 0.90 |
| KCCQ symptom burden score, mean ± SD | -1.86 (12.73) | -4.08 (14.47) | 0.33 | -1.19 (7.11) | 0.82 | -3.75 (11.30) | 0.54 |
| KCCQ self-efficacy score, mean ± SD | -2.27 (24.83) | 1.25 (30.54) | 0.45 | 1.79 (14.94) | 0.47 | -3.12 (14.55) | 0.88 |
| KCCQ quality of life score, mean ± SD | 0.58 (16.04) | 0.49 (21.24) | 0.98 | -0.99 (11.63) | 0.67 | -2.92 (19.55) | 0.39 |
| KCCQ social limitation score, mean ± SD | -0.73 (13.91) | -3.42 (16.13) | 0.36 | 0.81 (10.03) | 0.66 | -3.85 (19.35) | 0.49 |
| KCCQ total symptom score, mean ± SD | -2.21 (11.89) | -3.43 (13.38) | 0.57 | -0.69 (5.67) | 0.57 | -5.31 (11.47) | 0.29 |
| KCCQ clinical summary score, mean ± SD | -1.47 (9.19) | -4.61 (15.35) | 0.12 | 0.05 (7.09) | 0.48 | -5.70 (11.94) | 0.08 |
| KCCQ overall summary score, mean ± SD | -0.59 (8.89) | -3.24 (15.28) | 0.18 | -0.12 (5.83) | 0.82 | -4.29 (13.08) | 0.12 |
